# Cytomegalovirus Infection and Guillain-Barré Syndrome: The First Case-Control Study in Iran 

**DOI:** 10.22037/ijcn.v15i4.31285

**Published:** 2021

**Authors:** Setareh Mamishi, Mahmoud Reza Ashrafi, Mahmoud Mohammadi, Gholamreza Zamani, Babak Pourakbari, Shima Mahmoudi, Solmaz Aziz-Ahari

**Affiliations:** 1 1.Pediatric Center of Excellence, Children’s Medical Center. Tehran University Medical Science. Tehran. Iran; 2 2.Pediatric infectious Research Center. Tehran University Medical Science. Tehran. Iran

**Keywords:** Guillain-Barré syndrome, Cytomegalovirus, Children

## Abstract

**Objectives:**

Guillain-Barré syndrome (GBS) is an immune-mediated disease of the peripheral nervous system affecting all age groups around the world. Although the pathogenesis and optimal treatment of GBS have not yet been completely identified, one of the most common infectious diseases to trigger the syndrome is cytomegalovirus (CMV) infection. The GBS following CMV infection is rarely reported in childhood, and there have been no data on GBS with antecedent CMV infection in children in Iran. The current study aimed to evaluate the association between CMV infection and GBS in children in Iran.

**Materials & Methods:**

The case-control study design was used for 30 GBS cases and 30 matched controls. All the serum samples were tested for the presence of anti-CMV immunoglobulin M (IgM) and immunoglobulin G (IgG) antibodies using a commercially available enzyme-linked immunosorbent assay (EUROIMMUN Medizinische, Germany). The CMV viral deoxyribonucleic acid (DNA) in the specimen was detected using polymerase chain reaction (PCR) (Cytomegalovirus PCR Detection Kit, CinnaGen Co., Iran).

**Results:**

Anti-CMV IgG antibodies were detected in 97% of the GBS patients and 93% of the healthy controls. Anti-CMV IgM antibodies were demonstrated in 33% of the healthy controls (n=10) and 33% of the GBS children (n=10). The borderline level of anti-CMV IgM antibodies was observed in 23% of the healthy controls (n=7) and 13% of the GBS children (n=4) (P=0.57). None of the specimens from both controls and GBS cases was positive for CMV DNA using PCR.

**Conclusion:**

The obtained data demonstrated the presence of anti-CMV antibodies in the majority of both GBS patients and controls. Moreover, no relation was observed between CMV infection and GBS. However, it is highly recommended to perform further studies with a large sample size.

## Introduction

Guillain-Barré syndrome (GBS) is an immune-mediated disease of the peripheral nervous system occurring after an infectious disease in 60-70% of cases (1, 2). The GBS affects all age groups around the world; however, there has been a higher incidence in adults than that in children (3). The annual incidence of GBS in children is estimated at 0.34-1.34 cases per 100 000 individuals, with a slightly higher incidence in males (3, 4).

Although the pathogenesis and optimal treatment of GBS have not yet been completely identified (3), it has been reported that more than 70% of GBS patients have a history of a viral infection approximately 6 weeks before the development of the disease (3). Moreover, the possible role of some medications or procedures as probable etiologies has been reported (5). Some of the pathogenic triggers of GBS include viruses, such as Epstein-Barr virus, cytomegalovirus (CMV), hepatitis, varicella (6-9), *Campylobacter jejuni* (10, 11), and *Mycoplasma pneumoniae *(12). 

More recently, Zika virus and severe acute respiratory syndrome coronavirus 2 infections have also been reported as the potential triggers of GBS (2, 13, 14). Campylobacter jejuni enteritis (in 21-32% of cases) and primary CMV infection (in 10-22% of cases) have been reported as the most common infectious diseases to trigger the syndrome (7, 15). It has been reported that the risk of developing GBS following CMV infection is close to 1 in 1000 (7). However, the role of CMV infection in the prognosis of GBS is also still unclear (3, 8, 9).

There has been limited evidence on the epidemiology and prognostic factors of GBS following primary infection with CMV and the roles of anti-ganglioside antibodies, cellular immune responses, and viral replication (7). The GBS following CMV infection is rarely reported in childhood (16), and there have been no data on GBS with antecedent CMV infection in children in Iran. Therefore, the current study aimed to evaluate the association between CMV infection and GBS in children in Iran.

## Materials & Methods

This study was approved by the Ethics Board of Children’s Medical Center, Tehran University of Medical Sciences, Tehran, Iran. Furthermore, all the patients signed informed consent. The case-control study design was used for 30 GBS cases and 30 matched controls. The study was performed on GBS patients hospitalized in Children’s Medical Center, an Iranian referral hospital, for 2 years. The diagnosis of GBS was made according to the Asbury and Cornblath diagnostic criteria (17). The precise diagnosis of GBS Hughes functional classification scores at three different time points were calculated using clinical information available in the medical records. The Hughes functional classification score ranges from 0-6 as grade 0 (healthy), grade 1 (minor symptoms or signs, able to run), grade 2 (able to walk without assistance but unable to run), grade 3 (able to walk with assistance), grade 4 (bed- or chair-bound), grade 5 (requiring assisted ventilation for at least part of the day), and grade 6 (dead) (18).

All the serum samples were tested for the presence of anti-CMV immunoglobulin M (IgM) and immunoglobulin G (IgG) antibodies using a commercially available enzyme-linked immunosorbent assay (EUROIMMUN Medizinische, Germany) according to the manufacturer’s instructions. The CMV viral deoxyribonucleic acid (DNA) in the specimen was detected using polymerase chain reaction (PCR) (Cytomegalovirus PCR Detection Kit, CinnaGen Co., Iran).

**Data Analysis**

Data analysis was carried out using SPSS software (version 20.0; IBM Corp., Armonk, NY, USA). Frequencies and percentages were calculated for all the categorical variables. The Chi-square and t-test were applied for data analysis, and p-values of less than 0.05 were considered statistically significant. 

## Results

There were 30 children with GBS admitted to Children's Medical Center, an Iranian referral hospital, for 2 years. All the patients fulfilled the current diagnostic criteria for GBS. In addition, 30 controls were included in the study. The mean age values of the patients and controls were 6.5±3.8 and 6.6±3.9 years, respectively. There was no significant difference in age between the two groups. Overall, 60% of both groups were male. Moreover, 47% of the patients (n=14) were diagnosed during winter.

The median level of cerebrospinal fluid protein in children with GBS was 48 (interquartile range: 25-71) mg/dL. Preceding upper respiratory infection and diarrhea were observed in 70% (n=21) and 7% (n=2) of children with GBS, respectively. The severity of GBS, according to the Hughes scale, in the majority of the cases was at scores 4 (n=15; 50%) and 3 (n=11; 37%).

Anti-CMV IgG antibodies were detected in 97% of the GBS patients and 93% of the healthy controls (Table 1). Anti-CMV IgM antibodies were demonstrated in 33% of the healthy controls (n=10) and 33% of the GBS children (n=10). The borderline level of anti-CMV IgM antibodies was observed in 23% of the healthy controls (n=7) and 13% of the GBS children (n=4) (P=0.57). None of the specimens from both controls and GBS cases was positive for CMV DNA using PCR. 

**Table 1:** Comparison of Results of Polymerase Chain Reaction and Serological Findings of Cytomegalovirus Infection in Patients with Guillain-Barré Syndrome and Controls

Variable	GBS cases	Controls
Age, median (IQR), y	6.5±3.8	6.6±3.9
Gender (male), n (%)	18 (60)	18 (60)
CMV DNA present in serum, n (%)	0	0
CMV‐IgG seropositive, n (%)	29 (97)	28 (93)
CMV‐IgM seropositive, n (%)	10 (33)	10 (33)
CMV‐IgM borderline, n (%)	4 (13)	7 (23)

GBS, Guillain-Barré syndrome; IQR, interquartile range; CMV, cytomegalovirus; DNA, deoxyribonucleic acid; IgG, immunoglobulin G; IgM, immunoglobulin M

## Discussion

To the best of our knowledge, this has been the first study evaluating the association between CMV infection and GBS. Although there have been several reports about the serological findings of anti-CMV antibodies, there have been insufficient data on children. Moreover, there were no control groups in the majority of previous reports. 

This study evaluated the association between CMV infection and GBS in 30 GBS children and 30 healthy age- and gender-matched controls. It was demonstrated that there was no association between preceding CMV infection and GBS development. Similar to a previous report, GBS was more common in males than females (19). The incidence of GBS and its relation to seasonal variation was observed in the present study with predominance in the winter that is in contrast to the results of a previous study reporting months closely followed by spring (19).

In the current study, plasma CMV PCR test results were negative in all cases of both groups; however, in a study conducted by Orlikowski *et al.*, it was positive in 62% (36/58) of the patients with GBS (7). The present study did not identify CMV DNA in the patients with GBS that might suggest the geographical differences in the incidence of CMV-related GBS. Kuijf *et al.* reported only one case with positive CMV DNA (8); nevertheless, Steininger *et al.* identified the presence of CMV DNA in the serum of 33% of GBS patients (9). 

In the present study, anti-CMV IgM antibodies were demonstrated in 33% of the healthy controls (n=10) and 33% of the GBS children (n=10), which were higher than those of previous reports in children with GBS (20). None of the patients with positive CMV IgM serological test results had CMV DNA present in the serum that is consistent with the results of a study by Steininger *et al. *(9). In addition, in a study performed by Kuijf *et al.*, among the serum samples positive for CMV DNA, only 2 (15%) samples had CMV-specific IgM antibodies (8).

The results of the current study showed that 29 patients (97%) with GBS were CMV-IgG seropositive among whom 10 patients were CMV-IgM negative seropositive, indicating that the 20 patients (67%) with GBS were not in the acute CMV infection phase. However, CMV-specific IgM antibodies might be due to the reactivation of latent infection (15). The IgM antibodies were present in 30-50% of GBS patients who had CMV infection; however, IgM antibodies might be elevated in CMV infection without the development of GBS (21), which is observed in one-third of the patients and controls of the present study.

A high positive rate of CMV-IgG was observed in the serum of both cases and controls with no statistical significance. Therefore, it cannot be concluded that CMV is associated with GBS in children, and additional studies are needed to determine the relationship between GBS and antecedent infectious diseases.

The present study still has several limitations. First, this study had a small sample size. Second, CMV IgG avidity assays, as good methods for identifying the onset of primary CMV infection, were not performed in this study. The DNA of CMV can be detected several times during the infection. Since the anteceding infectious disease occurs about 6 weeks before GBS, negative PCR may not completely rule out the association between CMV infection and GBS; therefore, using the IgG avidity test could be beneficial for interpretation.

**In conclusion,** the obtained data demonstrated the presence of anti-CMV antibodies in the majority of both GBS patients and controls. Furthermore, no relation was observed between CMV infection and GBS. However, it is highly recommended to perform further studies with a large sample size.

## Author’s Contribution

Solmaz Aziz-Ahari,Setareh Mamishi,Mahmoudreza,Mahmoud Mohammadi,Gholamreza Zamani Ghaletaki were involved in the study design, Babak Pourakbari contributed data and analysis tools. Solmaz Aziz-Ahari collected the data and performed the analysis, Shima Mahmoudi**.**
